# A Case Report of a LVAD Driveline Infection Diagnosed by Point-of-care Ultrasound

**DOI:** 10.5811/cpcem.1403

**Published:** 2023-05-26

**Authors:** Nicholas Bielawa, Allison Cohen, Milan Patel, Brendon Stankard, Mathew J. Nelson

**Affiliations:** North Shore University Hospital, Manhasset, Department of Emergency Medicine, New York

**Keywords:** left ventricular assist device, driveline infection, abscess, point-of-care ultrasound, case report

## Abstract

**Introduction:**

As the prevalence of patients with left ventricular assist devices (LVAD) presenting to the emergency department (ED) increases, clinicians must be aware of LVAD-associated infections.

**Case Report:**

A well-appearing, 41-year-old male with history of heart failure status post prior-LVAD placement presented to the ED for swelling of his chest. What appeared initially as a superficial infection was further assessed with point-of-care ultrasound and found to represent a chest wall abscess involving the driveline, ultimately resulting in sternal osteomyelitis and bacteremia.

**Conclusion:**

Point-of-care ultrasound should be considered an important tool in the initial assessment of potential LVAD-associated infection.

## INTRODUCTION

Continuous-flow left ventricular assist devices (LVAD) have improved the survival of patients with end-stage systolic heart failure.^1^ Their complications include but are not limited to bleeding, device thrombosis, stroke, and infections.^2^,^3^ Of these complications, infections are less prevalent; however, they are the second most common cause of morbidity and mortality in patients who survive the initial six months after continuous-flow placement and are a leading cause of hospital admission in this patient population.^2^

According to the International Society for Heart and Lung Transplantation, LVAD infections are broken up into LVAD-specific infections, LVAD-related infections, and non-LVAD infections.^2^ Infections specific to LVADs include pump and/or cannula infections, pump pocket infections, and driveline infections (DLI), which are further broken down into superficial and deep infections.^2^ Related infections include infective endocarditis, bacteremia, and sternal wound/surgical site infections.^2^ The Interagency Registry for Mechanically Assisted Circulatory Support found a 19% prevalence in DLIs 12 months post-device implantation.^4^,^5^

It is difficult to discern the extent of the infection with only radiographic imaging. Computed tomography (CT) images are limited by artifact from the device. Point-of-care ultrasound (POCUS) can detect pockets of concern but cannot effectively inform whether deeper structures are involved.^4^ As a result, surgical exploration is often needed.^4^

## CASE REPORT

A 41-year-old Hispanic male with a history of American College of Cardiology/American Heart Association Stage D chronic systolic heart failure secondary to prior anabolic steroid use, status post HeartMate 3 LVAD placement three years prior to presentation, on warfarin, presented to the emergency department (ED) due to concern for midsternal chest wall swelling that developed spontaneously two days prior. The patient denied any trauma to the chest, fever, chills, or rash. The patient reported a small amount of pain to the area with palpation only, and no active drainage. Initial vital signs were significant for a mean arterial pressure of 62 millimeters of mercury, heart rate of 78 beats per minute, respiratory rate of 20 breaths per minute, oxygen saturation of 98% on room air, and oral temperature of 37.2 degrees Celsius (98.9° Fahrenheit). Physical examination revealed a 2 centimeter (cm) × 2 cm, elevated, mildly tender, midsternal collection without overlying erythema or crepitus. Laboratory results found a white blood cell count of 8.94 thousand per microliter (K/uL) (reference range: 3.8–10.5 K/uL), hemoglobin of 7.6 grams per deciliter (g/dL) (13–17 g/dL), platelet count of 279 (K/uL) (150–400 K/uL), and an international normalized ratio of 2.53 (0.88–1.16). Serum electrolytes were within normal limits.

**Image 1:**
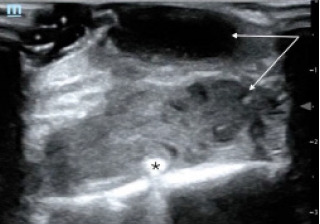
A point-of-care ultrasound view of the anterior chest wall, showing a complex fluid collection with irregular borders and mixed echogenicity (arrows) and surrounding inflammatory changes extending deep to the hyperechoic driveline (star).

A point-of-care ultrasound was performed to further evaluate the fluid collection. The POCUS revealed a complex, mixed echogenic, collection that extended deep to chest wall and appeared contiguous with the driveline wire (Image 1).

**Image 2:**
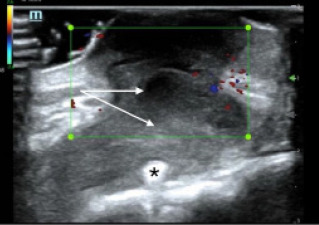
A point-of-care ultrasound of the anterior chest wall with color flow Doppler demonstrating a heterogeneous structure (arrows), extending to the driveline (star) without significant internal color flow.

Swirling of complex material was evident with compression. Use of color flow Doppler demonstrated an avascular heterogeneous structure, concerning for a deep space infection with driveline involvement (Image 2).

After POCUS was performed, a CT of the chest with intravenous contrast was ordered, and blood cultures were drawn due to concern for acute infection. The patient was given 2000 milligrams (mg) of cefepime and 1000 mg of vancomycin for antibiotic coverage. Computed tomography of the chest revealed a fluid-attenuating midline chest-wall lesion extending to the anterior sternal border measuring 2 cm × 1.9 cm × 3.4 cm with mild peripheral enhancement suggestive of a chest wall abscess.

*CPC-EM Capsule*

What do we already know about this clinical entity?

*Left ventricular assist device (LVAD) infections are a significant cause of morbidity and mortality; computed tomography diagnosis can be limited by artifact*.

What makes this presentation of disease reportable?

*To date, there have been no reports on the use of ultrasound to diagnose LVAD driveline infections*.

What is the major learning point?

*Point-of-care ultrasound should be considered an important tool in the initial assessment of potential LVAD driveline infections*.

How might this improve emergency medicine practice?

*Point-of-care ultrasound can allow for rapid detection of LVAD driveline infections, leading to earlier identification and management*.

While in the ED the patient developed a fever of 38.3° Celsius and became tachycardic. He was admitted to the cardiothoracic surgery service for definitive management. On admission, the fluid collection opened spontaneously and drained purulent material. A culture was sent and grew *Staphylococcus epidermidis*, and the first set of blood cultures grew *Streptococcus mitis/oralis*. The patient went to the operating room (OR) for irrigation and debridement of the sternal wound, removal of sternal wires except the inferior-most wire, and application of a wound vacuum-assisted closure device. Intraoperative transesophageal echocardiogram did not reveal valvular vegetations, and a wound culture obtained during debridement grew rare *Propionibacterium acnes*. The patient returned to the OR later in his hospital stay for further wound exploration and debridement. He was discharged on hospital day 17 on long-term antibiotic therapy with ceftriaxone 2000 mg via a peripherally inserted central catheter line.

## DISCUSSION

In this case, POCUS was essential for making the diagnosis of a deep space infection. What appeared on physical exam to be only a superficial pustule, was discovered on POCUS to be a deep soft tissue infection involving the driveline. Point-of-care ultrasound is a safe, quick, and accurate way for emergency clinicians to differentiate between cellulitis and abscess.^6^ Previous studies have shown the diagnostic utility of POCUS for skin infections by enhancing the clinician’s ability to distinguish an abscess from simple cellulitis, which can be difficult to evaluate on physical exam.^7^,^8^ Sonographic findings of an abscess include heterogeneity, often with irregular borders, that is largely anechoic or hypoechoic containing mixed echogenic foci, and displaying posterior acoustic enhancement.^9^ In an abscess with mixed echogenicity, gentle pressure can produce a “swirl” sign, or “ultrasonic fluctuance,” to help confirm the presence of purulent material.^9^ Additionally, the use of color flow Doppler is also helpful in evaluating suspected abscess by excluding the presence of vascular flow and demonstrating peripheral hyperemia.^9^

In this patient, a complex-appearing fluid collection was visualized extending deep into the chest wall and communicating with the driveline wires concerning for a driveline infection. The results of this POCUS exam allowed for rapid diagnosis and early antibiotic administration. Although CT is typically preferred to assess for deep LVAD infections, the sensitivity and specificity of CT for the detection of these LVAD infections are not well-defined given the lack of a gold standard test for comparison.^3^,^10^ On CT imaging, driveline infections may appear as rim-enhancing fluid collections containing gas pockets or soft-tissue stranding adjacent to the device components.^11^ However, this imaging modality may be limited by the interpreter’s familiarity with LVAD anatomy and pathology and significant artifact from the LVAD hardware.^10^

Point-of-care ultrasound allows for the rapid and accurate evaluation of skin and soft tissue infections at the bedside, and although there have been no reports to date on the use of POCUS to detect LVAD infections, prior research has shown that ultrasound is more sensitive than CT for diagnosing certain soft tissue abscesses and provides more details regarding the contents of the abscess cavity. 6–8,12 Other specialty imaging studies including leukocyte-labeled scintigraphy and positron emission tomography in combination with CT imaging may be more sensitive and specific for LVAD infection; however, cost, availability, and practicality in the ED limit the use of these modalities.^10^

This patient, who presented with superficial swelling of the chest wall, suffered from several infectious complications associated with his LVAD, including a DLI with associated chest wall abscess, sternal osteomyelitis, and LVAD-associated bloodstream infection (BSI). Driveline infections are the most frequent LVAD infection overall and occur most often within the first year of LVAD implantation^10^,^13^,^14^ Superficial DLIs spare the muscle and fascial layers while deep DLIs affect deeper structures, as was the case in this patient. Superficial DLIs have an unclear effect, while deep DLIs increase mortality in this group.^13^ The management of this patient involved a prolonged course of intravenous antibiotics and surgical debridement, which is often required in the treatment of deep LVAD infections.^4^,^10^,^13^ Clinicians should have a low threshold for evaluating and treating for possible LVAD infection in the ED, as the majority of these patients may not present with typical systemic inflammatory response syndrome in response to serious infections such as BSIs.^3^,^13^

## CONCLUSION

Overall, the rate of LVAD implantation has increased greatly in recent years, and as the prevalence of LVADs increases, so too will the prevalence of LVAD patients in the ED.^3^,^15^ Although advances in LVAD technology have reduced the rate of complications, infections such as deep DLIs and BSIs still account for significant morbidity and mortality in this group.^10^,^13^–^15^ It is imperative that emergency clinicians be aware of infectious complications associated with LVADs and how to efficiently and effectively manage patients with these devices.

This case presents an LVAD recipient with a DLI associated with a chest wall abscess, sternal osteomyelitis, and bloodstream infection. Point-of-care ultrasound provides a fast, portable, and accurate method of detecting soft tissue infections that may be missed on physical exam, and in this case detection of a fluid collection on ultrasound ultimately led to the diagnosis of serious infectious complications in an LVAD recipient.^6^–^8^,^12^ Point-of-care ultrasound was essential in making this initial diagnosis and should be considered an important tool in the initial assessment of potential LVAD driveline infection. Further research is necessary to determine the accuracy of POCUS for the diagnosis of certain LVAD-related infections such as driveline infections compared to CT.
